# Recent Insights into the Role of Herpesviridae in Alzheimer’s Disease: A Structured Narrative Review Based on a Systematic Literature Search

**DOI:** 10.3390/neurolint18070125

**Published:** 2026-06-29

**Authors:** Domenico Plantone, Carlo Manco, Delia Righi, Stefania Lago, Alessio Rocco Sangiorgio, Valentina Schino, Matteo Pardini, Angela Stufano, Guglielmo Lucchese

**Affiliations:** 1 Department of Medicine, Surgery and Neuroscience, University of Siena, 53100 Siena, Italy; domenico.plantone@unisi.it (D.P.); carlooo95.cm@gmail.com (C.M.); d.righi4@student.unisi.it (D.R.); s.lago@student.unisi.it (S.L.); a.sangiorgio@student.unisi.it (A.R.S.); 2 Interdisciplinary Department of Medicine, Section of Occupational Medicine, University of Bari Aldo Moro, 70100 Bari, Italy; valentina.schino@uniba.it; 3 Department of Neuroscience, Rehabilitation, Ophthalmology, Genetics, Maternal and Child Health (DINOGMI), University of Genoa, 16100 Genoa, Italy; matteo.pardini@unige.it; 4 IRCCS Azienda Ospedaliera Metropolitana, Polo Ospedale San Martino, 16100 Genoa, Italy; 5 Department of Medical and Surgical Sciences, University of Foggia, 71100 Foggia, Italy; angela.stufano@unifg.it; 6 Department of Experimental Medicine, University of Salento, 73100 Lecce, Italy; 7 Neurology Unit, “Vito Fazzi” Hospital, 73100 Lecce, Italy

**Keywords:** herpesvirus, dementia, cognition, cognitive impariment, proteinopathy

## Abstract

**Background/Objectives**: Herpesviridae have been increasingly investigated as possible contributors to Alzheimer’s disease (AD) because of their neurotropism, lifelong latency, and capacity to modulate inflammatory and amyloid-related pathways **Methods**: This structured narrative review, based on a systematic literature search, examined original studies published between December 2020 and December 2025 on the association between human herpesviridae and AD. Searches were conducted in PubMed, Web of Science, and Scopus using a PRISMA-informed screening framework. **Results**: The available evidence was heterogeneous across viruses and study designs. HSV-1 emerged as the most consistently implicated virus, supported by epidemiological, biomarker, neuroimaging, and experimental studies, although contradictory findings were also reported. VZV was mainly associated with AD through vascular, inflammatory, and vaccination-related evidence, with several studies suggesting lower dementia risk after zoster vaccination. CMV, EBV, and HHV-6 showed biologically plausible but less consistent associations, whereas HHV-7 and HHV-8 were supported only by limited or indirect evidence. Across studies, the proposed mechanisms included chronic neuroinflammation, vascular injury, amyloid and tau dysregulation, host immune responses, and cumulative infectious burden. **Conclusions**: Overall, the literature suggests that the relationship between herpesviridae and AD is not uniform and that HSV-1 appears to be the most relevant candidate. However, methodological heterogeneity, possible reverse causation, and unequal study intensity across viruses limit firm conclusions. More robust longitudinal and subtype-specific studies are needed to clarify causal relevance.

## 1. Introduction

The hypothesis of an association between pathogens, in particular viral ones, and neurodegeneration is not new, but it has gained momentum in recent years [1]. For instance, it has been known for decades that the Epstein–Barr virus (EBV) confers a higher risk of multiple sclerosis and autoimmune cross-reactions, and various molecular and immune-mediated mechanisms were investigated [2,3]. What is now considered to be definitive confirmation of such an association finally arrived in 2022 with basic research and epidemiological studies [2,4]. With specific focus on cognition, viral infections have been investigated as possible risk factors for dementia, specifically of Alzheimer’s type for years, and the SARS-CoV-2 pandemic has sparked even more interest in the possible mechanisms mediating increased cognitive risk from human–pathogen interaction [5,6,7,8].

Among the neurotropic viruses implicated in post-infectious long-term cognitive impairment and higher dementia risk, herpesviridae play a major role [9,10].

In the present work, we provide a structured narrative synthesis based on a systematic literature search of original research published during the last five years on the relationship between human herpesviridae—herpes simplex virus 1 (HSV-1), herpes simplex virus 2 (HSV-2), varicella-zoster virus (VZV), Epstein–Barr virus (EBV), Cytomegalovirus (CMV), human herpesvirus 6 (HHV-6), human herpesvirus 7 (HHV-7), and human herpesvirus 8 (HHV-8)—and Alzheimer’s disease (AD).

Compared with previous reviews, this article focuses specifically on recent original studies and explicitly separates epidemiological association, mechanistic plausibility, biomarker evidence, interventional evidence, and causal interpretability across viral subtypes.

## 2. Materials and Methods

### 2.1. Study Design and Reporting Framework

This work was conducted as a structured narrative review based on a systematic literature search aimed at synthesizing current evidence on the association between human herpesviridae and Alzheimer’s disease (AD). The search, screening, and reporting process was informed by the PRISMA 2020 (Preferred Reporting Items for Systematic Reviews and Meta-Analyses) framework to ensure transparent documentation of literature identification, screening, eligibility assessment, and inclusion. However, the objective of the review was qualitative and integrative rather than quantitative. Given the substantial heterogeneity in study designs, exposure definitions, AD diagnostic criteria, outcome measures, and inferential levels across the included literature, a narrative synthesis approach was adopted. Quantitative meta-analysis was not performed because the available studies did not provide sufficiently comparable effect measures, exposure definitions, and outcome definitions for meaningful pooling without risking misleading estimates.

### 2.2. Conceptual Framework and Research Objectives

The review was designed to address the following principal research domains: (1) the epidemiological evidence linking infection with individual human herpesviridae to the risk or prevalence of AD; (2) the neuropathological and molecular mechanisms proposed to mediate this relationship, including amyloid and tau dysregulation, neuroinflammation, vascular injury, and immune-mediated processes; (3) the evidence from biomarker and neuroimaging studies exploring herpesvirus-associated AD-relevant changes; and (4) data from antiviral and vaccination studies that may indirectly inform causal inference. The research questions were formulated using a modified PICOS framework:

Population: Human subjects of any age, including general population cohorts, clinical AD samples, and cognitively unimpaired individuals studied prospectively. Exposure: Infection with or seropositivity against any member of the Herpesviridae family, specifically herpes simplex virus types 1 and 2 (HSV-1, HSV-2), varicella-zoster virus (VZV), Epstein–Barr virus (EBV), cytomegalovirus (CMV), and human herpesviruses 6, 7, and 8 (HHV-6, HHV-7, HHV-8). Comparator: Uninfected or seronegative individuals, or comparator groups with lower viral burden, when available. Outcomes: Diagnosis of AD or dementia, AD-related neuropathological changes (amyloid burden, tau pathology), AD-associated biomarkers in blood or cerebrospinal fluid, neuroimaging findings, cognitive performance measures, and risk modification through antiviral or vaccination interventions. Study design: Original empirical research including epidemiological cohort, case–control, and cross-sectional studies; experimental studies in human subjects or human-derived models; Mendelian randomization analyses; and biomarker-based investigations. Secondary literature, including reviews, meta-analyses, editorials, letters, conference abstracts, and posters, was excluded from the primary synthesis.

### 2.3. Literature Search Strategy

A systematic literature search was conducted in three bibliographic databases: PubMed, Web of Science (WOS), and Scopus. Searches were restricted to studies published between December 2020 and December 2025. No language restriction was applied at the search stage, although studies not available in full text in a language readable by the review team were subsequently excluded at the eligibility stage. Search strings were adapted to the indexing syntax and controlled vocabulary of each database. The Boolean search strategy employed the following combination of terms:

(“Alzheimer Disease” OR “Alzheimer”) AND (“Herpesviridae” OR “Herpesvirus” OR “Herpes Simplex Virus 1” OR “Herpes Simplex Virus 2” OR “Varicella-Zoster Virus” OR “Epstein-Barr Virus” OR “Cytomegalovirus” OR HSV-1 OR HSV1 OR “herpes simplex virus 1” OR “herpes simplex 1” OR HSV-2 OR HSV2 OR “herpes simplex virus 2” OR “herpes simplex 2” OR “VZV” OR “varicella zoster” OR “varicella-zoster” OR shingles OR “EBV” OR “Epstein Barr” OR “Epstein-Barr” OR “CMV” OR “cytomegalovirus” OR “HHV-6” OR “HHV6” OR “human herpesvirus 6” OR “HHV-6A” OR “HHV-6B” OR “HHV-7” OR “HHV7” OR “human herpesvirus 7” OR “HHV-8” OR “KSHV” OR “Kaposi sarcoma herpesvirus”) NOT (“Review” OR “Systematic Review” OR “Meta-Analysis” OR “meta-analysis” OR “metaanalysis”).

Reference lists of eligible articles and relevant narrative or systematic reviews identified during screening were manually checked using a snowball approach to identify any additional studies not retrieved by the primary database search.

### 2.4. Study Selection and Eligibility Criteria

All records retrieved through the database searches were imported into Rayyan (https://www.rayyan.ai/, accessed on the 3 January 2026), a web-based screening platform used for systematic review data management. Duplicate entries were identified and removed prior to screening. The selection process followed a sequential, three-stage approach in accordance with the four-phase PRISMA framework (Identification, Screening, Eligibility, and Inclusion). In the initial phase, titles were screened to exclude records clearly outside the scope of the review. Subsequently, abstracts were evaluated against the predefined eligibility criteria. Full-text articles were then retrieved and assessed in detail to confirm inclusion. All authors worked independently at each screening stage, and any discrepancies were resolved by consensus.

Although the review was structured according to a modified PICOS framework, not all included studies fulfilled each PICOS element in a strict comparative sense. In particular, while most studies met the population, exposure, and outcome criteria, several mechanistic, biomarker, genomic, in silico, or human-derived experimental studies did not include a conventional unexposed or seronegative comparator group. These studies were retained when they provided original empirical or mechanistically relevant evidence on Herpesviridae infection, serological response, viral detection, or virus–host interactions in relation to AD-related outcomes. Accordingly, the comparator criterion was applied where relevant and available, whereas non-comparative studies were interpreted as supportive or hypothesis-generating evidence rather than as direct epidemiological evidence. Both positive and null findings were eligible for inclusion. Studies were excluded if they: (1) consisted of narrative or systematic reviews, meta-analyses, editorials, letters, conference abstracts, or posters without original data; (2) did not include any AD-related clinical, biomarker, or neuropathological outcome; (3) were not available in full text; or (4) were published outside the predefined timeframe.

### 2.5. Data Extraction and Synthesis

For each included study, relevant information was extracted using a predefined approach. Extracted data included: publication details (authors, year, journal); study design and recruitment period; geographical setting; characteristics of the study population (sample size, age range, clinical status); herpesvirus species investigated and method of exposure ascertainment (serology, PCR, genomic analysis); AD or dementia diagnostic criteria; outcomes reported (clinical diagnosis, biomarker levels, neuroimaging findings, cognitive performance, risk estimates); and principal results. Data extraction was performed independently by at least two authors, and discrepancies were resolved by discussion.

The evidence was synthesized qualitatively and organized by herpesvirus species. Within each subsection, studies were grouped thematically according to their primary evidentiary contribution: epidemiological association studies; mechanistic and biomarker evidence; neuroimaging-based findings; antiviral and vaccination data; and null or contradictory results. This thematic structure was adopted to facilitate cross-study comparison and to highlight convergent, divergent, and inconclusive findings within each viral subtype.

To make heterogeneity explicit, included studies were additionally classified according to viral subtype, inferential level (association, null association, interventional evidence, Mendelian randomization, or experimental/mechanistic evidence), outcome domain (clinical diagnosis, cognitive performance, neuroimaging, fluid biomarker, tissue biomarker, or molecular pathway), and model system (human cohort, post-mortem human tissue, animal model, organoid/in vitro model, or in silico analysis). This classification was used to avoid giving equal causal weight to fundamentally different forms of evidence and is summarized in Table 1.

### 2.6. Methodological Quality Appraisal

A formal risk-of-bias scoring tool such as NOS, ROBINS-I, or Cochrane RoB was not applied because the included literature encompassed highly diverse study designs, including population-based observational cohorts, retrospective health-record analyses, Mendelian randomization studies, neuroimaging and biomarker studies, post-mortem investigations, animal experiments, organoid and in vitro studies, and in silico analyses. Applying a single scoring instrument across this evidence base would have created an artificial impression of comparability. Instead, methodological quality and interpretability were critically appraised narratively throughout the synthesis. Particular attention was given to herpesvirus exposure ascertainment (e.g., IgG serology versus PCR-based viral detection versus genomic analyses), possible exposure misclassification, AD or dementia ascertainment, confounder control including age, sex, APOE genotype, comorbidities, and medications, temporal ordering of exposure and outcome, reverse causation, sample size, and statistical power. These considerations informed the interpretation of each viral subtype and were explicitly incorporated into the discussion of evidence strength and remaining gaps.

**Figure 1:**
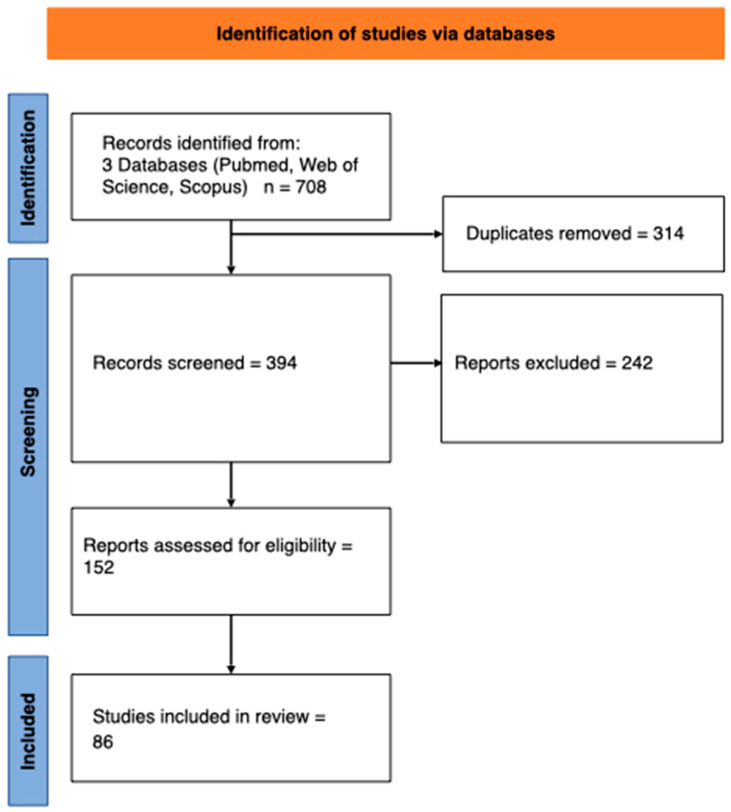
PRISMA flow diagram of study selection. Records were identified through searches of PubMed, Web of Science, and Scopus. A total of 708 records were retrieved, of which 314 duplicates were removed. After screening 394 records, 242 reports were excluded. The remaining 152 reports were assessed for eligibility, and 86 studies were included in the final review.

The search and selection process is shown in Figure 1. The included studies are presented in Table 2.

## 3. Results

### 3.1. The Role of Herpes Simplex Virus in Alzheimer’s Disease Pathogenesis

Herpes simplex virus type 1 (HSV-1) is a widespread neurotropic virus capable of reaching the brain and causing rare but severe herpes simplex encephalitis (HSE), which carries a high mortality rate [87]. Herpes simplex virus type 2 (HSV-2), though primarily associated with genital infections, can also cause encephalitis, often with severe neurological consequences [88].

The potential role of HSV-1 and HSV-2 in the pathogenesis of late-onset Alzheimer’s disease (AD) has been explored in numerous independent studies. The studies reviewed in this section can be categorized into three main groups: (1) studies investigating associations between HSV infections and AD prevalence; (2) studies examining underlying pathogenic mechanisms; and (3) studies assessing the effects of antiviral treatments.

#### 3.1.1. Association Studies: Positive Findings

Studies investigating associations between HSV infections and AD prevalence can be further distinguished between those that find positive associations and those that find no association. Beginning with studies that found positive associations, Vestin et al. [11] demonstrated that seropositivity for anti-HSV immunoglobulin G (IgG) is associated with more than double the risk of developing dementia. In this study, no significant interaction was found between HSV and apolipoprotein E ε4 (APOE ε4) or cytomegalovirus (CMV).

Linard et al. [12] documented that hippocampal volume reduction was only evident in subjects with high IgG levels, suggesting frequent viral reactivation. In APOE4 carriers, HSV-1 increased the risk of AD and further reduced hippocampal volume, while no effects were observed in non-carriers.

Liu et al. [13] analyzed data from 344,628 case–control pairs from the IQVIA PharMetrics Plus database (2006–2021), highlighting an association between symptomatic HSV-1 infection and AD risk, with a more pronounced effect in individuals over 75.

Shim et al. [14] demonstrated an increased risk of dementia (including AD, vascular dementia and Lewy body dementia) in patients with HSV-1 and varicella-zoster virus (VZV) infections using data from a national Korean cohort. Interestingly, HSV-1 subtypes were associated with dementia, while HSV-2 was not. Among the pathogenic mechanisms, the study hypothesized that HSV-1 induces amyloid-β (Aβ) accumulation and tau phosphorylation in neuronal cells, while VZV may hinder Aβ degradation by binding to insulin-degrading enzyme (IDE).

A retrospective study [15] of 33,236,890 patients aged ≥65 years analyzed the association between HSV infection (HSV-1, HSV-2 or coinfection) and the risk of developing dementia using data from electronic medical records from the TriNetX database. The results showed that all groups with a history of HSV (generic HSV, HSV-1, HSV-2 or coinfection) had a significantly higher risk of dementia compared to controls. Only males with HSV-2 showed a significantly higher risk of dementia compared to females.

Shin et al. [16] also documented that HSV-1/2 and VZV increase the risk of dementia, with coinfection (HSV + VZV) showing the highest risk and the shortest time between infection and dementia diagnosis. The study also found that ocular/CNS infections with VZV present a higher risk compared to simple forms.

Cantero et al. [17] investigated the association between HSV-1 and Aβ load in cognitively normal older adults, finding that increased anti-HSV IgG was correlated with higher Aβ load in frontotemporal regions, particularly in the anterior cingulate cortex, with effects moderated by APOE4 (the main genetic risk factor for AD). Anti-HSV IgG levels were not correlated with subclinical cognitive deficits or blood markers of neurodegeneration, suggesting a selective role of HSV-1 in Aβ deposition in normal aging.

An important epidemiological study conducted on the UK Biobank database [18] demonstrated that HSV-1 seropositivity is associated with an increased risk of dementia, while the combination of seropositivity for four neurotropic viruses (HSV-1, human herpesvirus 6 (HHV-6), HHV-7 and VZV) further amplifies this risk. The analysis, conducted on 9431 participants with serological data for 15 viruses, revealed that only HSV-1 showed a significant individual association, while the cumulative effect of the four neurotropic viruses suggests a synergistic role in the pathogenesis of dementia.

A genomic study [19] of 37,000 participants, conducted by analyzing blood samples (25,072) and brain samples (3449) through whole exome/genome sequencing (WES/WGS), found through machine learning analysis and logistic regression that HSV-1 DNA is significantly associated with the risk of AD.

A longitudinal study [20] of 2997 participants from the National Health and Nutrition Examination Survey III (NHANES III, 1988–1994), with follow-up until 2018 and documented HSV-1/2 seropositivity through enzyme-linked immunosorbent assay (ELISA) tests on serum samples, showed that only HSV-2 (hazard ratio (HR) = 1.30, *p* = 0.028), and not HSV-1, was associated with an increased risk of dementia.

The reduced seroprevalence of HSV-1 in a recent population-based cohort study in Sweden [21] suggests a potential increase in the risk of primary infections in adulthood, with possible implications for AD pathogenesis related to late viral reactivations and altered innate immune response.

#### 3.1.2. Alternative Perspectives on Chronic Infection

Some studies focus on the role of chronic infection and pathogen synergy in promoting neurodegeneration rather than the specific role of HSV-1 and HSV-2. For example, the study by Rangel et al. [22] examined the association between exposure to infectious pathogens (including HSV-1 and HSV-2) and cognitive decline in a cohort of elderly Hispanics. The analysis of 165 participants found that HSV-1 seropositivity did not show significant differences between groups, while cumulative exposure to multiple pathogens (including HSV-1 and HSV-2) was associated with an increased risk of cognitive impairment.

Roberts et al. [23] found an association between HSV-2 seropositivity and reduced cerebral cortical thickness in cognitively normal older adults. Analysis of 455 participants from the Northern Manhattan Study (NOMAS) showed that high levels of anti-HSV-2 antibodies correlate with a thinner cortex, while *Chlamydia pneumoniae* and HSV-2 together have an additive effect. HSV-2, through periods of latency and reactivation, could promote chronic neuroinflammation, accelerating brain aging. The lack of association with the AD signature suggests a more general impact on brain health rather than specific AD pathology. The relationship with C. pneumoniae indicates a possible synergy between infections in damaging cortical tissue, perhaps through common inflammatory pathways.

#### 3.1.3. Contradictory Evidence

Several studies have shown equivocal or null associations between HSV seropositivity and the development of neurocognitive disorders. Lopatko Lindman et al. [24] examined the relationship between plasma Aβ levels and antibodies against HSV-1, CMV and Chlamydophila pneumoniae in a large sample of AD patients and healthy controls. The results showed no significant correlation between Aβ_40_ and Aβ_42_ levels and the presence or levels of IgG antibodies against these pathogens, even after adjustment for confounding variables such as age and sex. Although AD patients showed higher HSV seroprevalence than controls, no differences in Aβ levels were observed.

Lapeyre et al. [25] investigated whether HSV-1 infection could accelerate Aβ pathology in a murine model of AD (APP/PS1). The results showed that intranasal infection with a neurovirulent strain of HSV-1 did not increase Aβ plaque load in the brains of APP/PS1 mice, nor did it alter microglia recruitment or circulating monocyte populations. No significant differences were observed in the number and volume of Aβ plaques or in the number of microglia between infected and uninfected mice. Furthermore, infection did not affect circulating monocyte subtypes, suggesting that HSV-1 does not exacerbate Aβ pathology in this model.

Bocharova et al. [26] examined the link between HSV-1 infection and β-amyloid (Aβ) pathology in murine models of AD. In 5XFAD mice (with pre-existing Aβ plaques), infection did not improve survival or reduce viral spread, suggesting that plaques do not protect against the virus. In a model of late-onset AD (hAβ/APOE4/Trem2*R47H), HSV-1 did not induce Aβ aggregation, even after lipopolysaccharide (LPS) stimulation or intracerebral inoculation. Histological analysis revealed that infection was primarily counteracted by infiltrating myeloid cells (not resident microglia), with no evidence of association between HSV-1 and Aβ.

Analysis of trigeminal ganglia samples (site of viral latency) and brains of patients with HSE found no evidence that latent or lytic HSV-1 infection directly induces aberrant expression of Aβ or phosphorylated tau (pTau) in infected neurons [27]. Key findings showed that while some HSE patients exhibited intraneuronal Aβ accumulation or extracellular plaques, these were not specific to HSV-1-infected areas nor correlated with viral presence. Even in rare cases where HSV-1 and AD pathology coexisted (e.g., a patient with concurrent AD and HSE), amyloid plaques and neurofibrillary tangles did not preferentially localize to viral replication sites. Furthermore, the inflammation associated with HSE, though marked, did not specifically influence Aβ or pTau expression.

Finally, a study on 5XFAD mice [28], which overexpress a mutated human form of amyloid precursor protein (APP) when infected with HSV-1 (strains 17syn+ and McKrae, at three different doses), demonstrated no protection of mice from infection. However, the 5XFAD mice showed HSV-1 replication centers partially excluded from brain areas with high densities of Aβ aggregates, and the Aβ aggregates themselves were devoid of HSV-1 viral particles. The limited viral invasion into areas with high Aβ density was attributed to the phagocytic activity of reactive microglia.

#### 3.1.4. Potential Therapeutic Effects of Antiviral Drugs

From the perspective of possible therapeutic effects of antiviral drugs to slow cognitive decline, valacyclovir showed no efficacy in slowing cognitive decline or modifying AD biomarkers in HSV-seropositive patients [29]. Antiviral treatment did not show a significant reduction in dementia risk, suggesting that the presence of IgG antibodies, rather than the frequency of reactivations, may be a risk indicator [11].

Tan et al. [30] analyzed data from the Australian Pharmaceutical Benefits Scheme (2013–2022) to assess the association between antiviral treatment for HSV/VZV and the dispensing of dementia medications in adults over 60. The results showed a 27% reduction in the risk of dementia medication dispensing among patients treated for herpes virus, with significant associations also found in longitudinal models.

Liu et al. [13], previously discussed for their positive association findings, highlighted that antiviral use reduced the risk of developing AD by 17%.

In the previously cited study by Sundstrom et al. [31] on the effects of HSV-1 infection on human brain organoids and pancreatic islets derived from stem cells, it was documented that treatment with acyclovir did not fully restore altered genes in pancreatic models, suggesting greater efficacy in countering neurological inflammation.

Finally, in another previously cited study [32] on brain organoids derived from pluripotent stem cells, it was demonstrated that treatment with antiviral drugs such as ribavirin and valacyclovir inhibits HSV-1 replication and restores AD-associated neuropathological phenotypes in infected brain organoids, suggesting potential therapeutic value in slowing AD progression.

### 3.2. Varicella-Zoster Virus (VZV) and Alzheimer’s Disease

Recent literature on VZV and AD can be grouped across a few overlapping aspects of the relationship between the pathogen and the disease, ranging from direct molecular mechanisms to population-level risk and prevention. Whether VZV can induce AD-like pathology or biomarker changes, whether VZV infection is associated with later cognitive decline or dementia, whether vaccination or antiviral treatment modifies that risk, and whether vascular or inflammatory pathways provide a plausible mechanistic bridge between infection and neurodegeneration are the main questions addressed.

#### 3.2.1. Mechanistic Links Between VZV and AD-like Pathology

Support for a biological relationship between the varicella-zoster virus (VZV) and Alzheimer’s disease (AD) comes from studies that place VZV within the machinery of amyloidogenesis, tau-related signaling, and vascular injury. Bubak et al. [33] show that VZV vasculopathy is accompanied by increased cerebrospinal fluid (CSF) amyloid, with amyloid levels correlating positively with amylin and anti-VZV antibodies, while VZV-infected vascular-associated cells accumulate intracellular amylin, Aβ42, and amyloid and generate a secreted environment capable of promoting amyloid fibril formation. In a complementary vascular-cell model, Bubak et al. [34] report that VZV-infected human brain vascular adventitial fibroblasts display transcriptional enrichment not only for vascular remodeling and extracellular-matrix disruption but also for pathways linked to amyloidosis, tauopathy, and Alzheimer disease, with confirmatory evidence of amyloidogenic peptides and intracellular amyloid in infected cells. Shinomoto et al. [35] add a related but distinct point by showing that acute HSV- and VZV-related CNS infection can produce CSF biomarker profiles that resemble AD, implying that herpesvirus-driven neuroinflammation may transiently recapitulate part of the AD biochemical signature. At the more speculative end, Serwer and Wright [89] propose that dementia may arise partly through maladaptive innate-immune protein assembly induced by viral infection, with VZV included as a plausible trigger in that framework. In sum, these studies suggest a potential role of VZV not only in inflammatory processes but also in AD-related amyloid and tau pathology.

#### 3.2.2. Biomarker Overlap and Preclinical Biomarker Acceleration

A second line of recent research focuses on exposure to VZV among other herpesviridae as a factor affecting AD-related biomarkers. Shinomoto et al. [35] show that VZV-associated CNS infection can generate CSF profiles that mimic AD, suggesting that acute infection may transiently affect amyloid and tau-related measures in ways that resemble neurodegeneration. On the other hand, Eckerström et al. [36] show that although patients remain cognitively impaired years after VZV encephalitis, they do not show convincing evidence of persistent AD-type CSF pathology, arguing against a simple equivalence between post-VZV cognitive sequelae and ongoing Alzheimer biology. Extending the herpesvirus question into aging, in the absence of clinical signs, James et al. [37] report that seropositivity to human herpesviruses is associated with steeper age-related increases in serum Aβ40, Aβ42, pTau181, and pTau217 in cognitively unimpaired women. Although the role of VZV could not be isolated, the study still supports the broader idea that prior herpesvirus exposure may accelerate trajectories of dementia-related biomarkers. Taken together, these works suggest that herpesvirus exposure, including acute VZV CNS disease, can intersect meaningfully with biomarker systems commonly used to infer AD-related pathology while also showing that such overlap does not automatically imply sustained Alzheimer-type neurodegeneration.

#### 3.2.3. VZV Infection as a Risk Factor for Dementia and AD

Two large recent Korean studies provide evidence for an association between VZV and risk of dementia: Shim and colleagues [14] report that VZV infection is associated with increased subsequent AD risk after multivariable adjustment, while Shin et al. [16] similarly find that both HSV and VZV are associated with an elevated risk of dementia, including AD and vascular dementia, and further show that ophthalmic, CNS, and complicated VZV infections are associated with higher risk than simple VZV infection. However, Lophatananon et al. [38] find only a small non-significant increase in dementia risk among people with shingles, even though the vaccination component of the same study points to a protective effect (see dedicated section below).

The broader literature focusing on infection burden including VZV is also not unequivocal. Wennberg et al. [39] emphasize the cumulative burden of common infections rather than a clean VZV-specific effect, and the subsequent correction explicitly notes that an earlier association between VZV and Mini-Mental State Examination (MMSE) is no longer significant after correction. Similarly, Mekli and colleagues [18] identify a stronger dementia signal when VZV is considered as part of a multi-virus neurotropic profile with HSV-1, HHV-6, and HHV-7 rather than as a standalone driver. Choi et al. [40] provide contrasting evidence, concluding that herpes zoster does not increase the risk of neurodegenerative dementia in their Korean case–control study. In sum, this body of work supports the possibility that VZV may increase the downstream risk of dementia and AD in at least some populations and clinical contexts, especially in the case of severe infection with primary neurological involvement, but the association is not uniformly replicated across study designs.

#### 3.2.4. VZV-Vaccination Evidence and the Question of Mechanism

The recent notion that VZV-vaccination might exert a neuroprotective effect is among the most compelling indirect evidence linking VZV biology to dementia risk, though it also raises the possibility that the effect is not entirely pathogen-specific. Lophatananon and colleagues [38] report that Zostavax vaccination is associated with lower dementia risk in the UK Biobank. A follow-up study [43] on data obtained from the United Kingdom Clinical Practice Research Datalink (CPRD) confirmed that zoster vaccination is associated with reduced risk of dementia overall and a lower hazard of AD specifically. Further evidence in this direction comes from the work of Xie et al. [44]. The authors use a birth-date-based eligibility threshold and conclude that herpes zoster vaccination appears to reduce the incidence of dementia and mild cognitive impairment as well as deaths due to dementia. These results suggest a benefit from VZV vaccination across multiple stages of the disease course. Additional similar evidence originates from the study by Lehrer and Rheinstein [42] on data from the United States Behavioral Risk Factor Surveillance System (BRFSS). At the same time, Harris et al. [45] complicate pathogen-specific interpretations by showing that lower AD risk is associated not only with herpes zoster vaccination but also with Tdap/Td and pneumococcal vaccination, raising the possibility that reduced dementia risk may partly reflect broader immune training, healthcare behavior, or reduced inflammatory burden rather than specific prevention of VZV reactivation. Thus, the vaccination literature supports the idea that preventing shingles or altering vaccine-induced immune responses is associated with better cognitive outcomes in older adults, but it leaves open whether the mechanism is primarily VZV-specific.

#### 3.2.5. Antiviral Treatment as Indirect Support for Association Between VZV Infection and AD Risk

Compared with the vaccination literature, treatment-based evidence is more limited, but it is conceptually important because it offers an intervention-oriented way of assessing whether herpesvirus activity may be relevant to dementia outcomes. Tan et al. [30] analyze Australian Pharmaceutical Benefits Scheme data and find that exposure to antiviral treatment for HSV/VZV infection is consistently, though not conclusively, associated with lower dispensing of antidementia medications. This does not establish that VZV causes AD, and the outcome is a prescribing proxy rather than adjudicated clinical dementia, but the study nonetheless strengthens the plausibility of a non-bystander role for herpesvirus infection. In other words, if treatment of herpesvirus infection tracks with lower downstream dementia-related medication use, then the infectious exposure may be more than a coincidental correlate of aging or frailty. This treatment literature remains more tentative than the vaccine evidence, but it helps reinforce the broader argument that reducing herpesvirus burden may influence later cognitive outcomes.

#### 3.2.6. Vascular and Inflammatory Pathways Linking VZV to AD-Relevant Injury

A particularly coherent mechanistic theme in this literature is that VZV may contribute to dementia and AD-related injury through the cerebrovascular and inflammatory interface rather than, or in addition to, classical plaque and tangle pathways. Bubak et al. [33] depict VZV vasculopathy as a setting in which persistent arterial inflammation is accompanied by amyloidogenic changes, suggesting that infection-driven vascular inflammation and amyloid deposition may amplify one another. Bubak et al. [34] extend that model by showing that infected vascular fibroblasts exhibit hallmarks of pathological vascular remodeling, including altered migration and matrix-linked signaling, alongside enrichment for AD-, amyloid-, and tau-related pathways. Shinomoto et al. [35] contribute complementary evidence that CNS herpes infections are accompanied by biomarker changes consistent with glial activation and neuroinflammation. Yeh et al. [41], while epidemiologic rather than mechanistic, explicitly frame herpes zoster as a potential source of neuroinflammation, cerebral vasculopathy, and direct neuronal injury, which fits closely with the mechanistic implications of the vascular studies. Read together, these papers support a model in which VZV may influence AD risk or expression through chronic inflammatory stress, cerebrovascular damage, and disturbance of neuroimmune homeostasis.

#### 3.2.7. Broader Herpesvirus and Neurodegeneration Context

Finally, several papers are useful less because they prove a specific VZV–AD relationship than because they situate VZV within a wider herpesvirus-neurodegeneration framework. Duggan et al. [53] connect viral antibody profiles to proteomic signatures and identify IGDCC4 as a mediator linking host antiviral immune responses to neurodegenerative outcomes, thereby illustrating one route through which systemic viral immunity could feed into dementia biology. Li et al. [90] similarly show in human neurons that infection with several endemic neurotropic viruses, including VZV, induces proteomic changes converging on pathways relevant to neurodegeneration and overlapping with alterations observed in AD brains. Taheri et al. [46] place VZV inside a broader inflammatory-microbial model, reporting increased expression of cytokines such as TNFα, IFNγ, TGFβ, and IL-10 in the presence of VZV and other microorganisms in Alzheimer and non-Alzheimer patients, while acknowledging VZV’s relevance chiefly through its neurotropic and immunostimulatory properties. James et al. [37] are also relevant here because they support a broader human-herpesvirus contribution to dementia-related biomarker acceleration, even though HHV-3/VZV could not be tested separately. These studies matter because they show that VZV is part of a larger ecological and mechanistic conversation about chronic viral exposure, immune response, and neurodegeneration, while also underscoring that the specificity of VZV’s role relative to other herpesviruses remains unresolved.

### 3.3. Epstein–Barr Virus (EBV) and Alzheimer’s Disease

Recent literature mostly focuses on the link between Epstein–Barr Virus (EBV) and multiple sclerosis (MS). In this paragraph, we aimed to provide an overview of the potential association between EBV and AD. Particularly notable is that elevated pathogen antibody titers are found to increase the risk of dementia/AD [91].

#### 3.3.1. Mendelian Randomization Evidence Linking EBV and AD

Zhang and colleagues performed a Mendelian randomization analysis (MR) using the inverse variant-weighted method to investigate the link between antibody-mediated immune responses to infectious disease agents and the risk of AD [47]. With the same method, Huang et al. provided evidence supporting a causal link between EBV infection and AD [48]. A complementary MR analysis by Peng et al. further investigated the association between EBV and increased AD risk, aligning with prior evidence [49].

#### 3.3.2. Anti-EBV Immune Responses and Cognitive Decline

Carbone et al. demonstrated that the duration of the EBV incubation period may exacerbate systemic immune responses and induce alterations in inflammatory processes, thereby contributing to age-related cognitive decline. Results show that the EBV nuclear antigen-1 (EBNA-1) antibody acted as a risk factor for AD, while the varicella-zoster virus glycoproteins E and I antibodies act as protective factors [50]. Similarly, K. Sim et al. showed significant down-regulation of the antibody anti-DG#29 targeting an epitope of EBNA1. This down-regulation increased AD risk in female patients and was associated with EBV reactivation in AD [51]. Regarding the female population, James and colleagues observed an association between HHV, most notably EBV, increasing serum biomarkers for dementia in MCI women [37]. Duggan et al.’s MR analysis revealed associations between coronavirus and herpesvirus antibody titers and dementia prevalence, cognitive performance, brain volumes, dementia biomarkers, and the plasma proteome. In particular, both EBNA-1 and EBV capsid antigen (VCA) show higher concentration levels linked to lower Aβ42/40 ratios, with EBNA-1 being associated with higher cortical amyloid, while VCA antibodies were related to lower frontal lobe volume [53]. In contrast, Murdock et al. found that antibody titers to the viral capsid antigen were not associated with executive function or circulating/stimulated inflammation in linear regression analyses [54].

#### 3.3.3. Molecular Mechanisms: ApoE Interaction, Amyloidogenesis, and Neuroinflammation

Tiwary and colleagues examined the interaction between EBV proteins, in particular EBNA-1 and BamHI Z Epstein–Barr virus Leftward Frame 1 (BZLF-1) with ApoE3. Their findings appear to suggest that the interaction between the two proteins and the CTD binding region of ApoE3 might interfere with ApoE’s amyloid-b binding capability, preventing its normal functioning [55]. Notably, their research identifies the peptide EBV-gM_146–157_, which exhibits aggregation properties similar to those of Aβ_42_, demonstrating a cytotoxic effect on neuroblastoma cells. The study supports the hypothesis that EBV may contribute to the formation of amyloid plaques in the brain [56]. Shifting the focus onto neuroinflammation, Patra et al. demonstrated that Glycoprotein M_146–157_ upregulates pro-inflammatory cytokines (IL-1β, IL-6, TNF-α, TGF-β) in neuronal cells [57]. On the therapeutic front, the same research group also proposed an intervention based on the inhibition of the activity of the viral enzyme dUTPase using phytochemicals, with the aim of disrupting EBV maintenance and reactivation by targeting its replication cycle. Through in silico analysis, Dehydroevodiamine (DHE) was identified as a promising candidate due to its effective binding to EBV-dUTPase, which is primarily mediated by electrostatic interactions. In detail, the protein–ligand binding occurs in the region surrounding His71, positioning DHE as a potential lead compound for future drug development against EBV, with the authors suggesting a potential neuroprotective effect [58]. Another in silico study aimed at identifying shared “hub genes” associated with both EBV infection and AD [59]. The authors identified through microarray analyses three miRNAs (hsa-miR-15a-5p, hsa-let-7a-5p, hsa-miR-7-5p) regulating hub genes such as YWHAH, YWHAG, YWHAB, YWHAZ, MAP2K1, PPP2CA, and TUBB, mostly belonging to the 14-3-3 family of proteins.

#### 3.3.4. Cohort and Population-Based Evidence

Wennberg et al.’s cohort study investigated the association between common viral infections (HSV-1, CMV, EBV, VZV, and TOX) and cognitive decline in 575 adults from the Baltimore ECA cohort. The results revealed that a higher seropositivity concentration level (particularly for CMV and HSV-1) correlated with poorer cognitive performance (as measured by MMSE), while suggesting that EBV may contribute to cognitive decline through chronic immune activation and neuroinflammation [39]. Malashenkova et al. studied the genetic infection rate of HHV, as well as their possible relationship with the level of the key cytokines and with clinical parameters of AD in patients with early and late onset [60]. Similarly, Mekli and colleagues found a positive association between EBV IgG antibody concentration levels and dementia risk [18,92]. This finding was validated by Levine et al. using FinnGen and UK Biobank data, which confirmed the link between EBV and dementia [61].

#### 3.3.5. Systems Biology, Viral Reactivation, and Co-Infection Patterns

Using systems bioinformatics, Onisiforou and Zanos demonstrated how Herpesviridae can influence cellular homeostasis dysfunction and microglial activation, specifying their potential role in the AD KEGG pathway, neuronal death and autophagy, oxidative stress response, and Aβ formation. Notably, their work highlights how the reactivation of herpesviruses such as HSV-1, CMV, and EBV during acute SARS-CoV-2 infection may potentially increase risk factors for neurodegeneration [52]. Interestingly, in the study conducted by Nemergut et al., they investigated the prevalence of the 10 most common viruses, HSV-1, EBV, HHV-6, HHV-7, CMV and bacteria (*Chlamydia pneumoniae*, *Helicobacter pylori*, *Borrelia burgdorferi*, *Porphyromonas gingivalis*, and *Treponema* spp.) in the serum, CSF, and brain tissue of AD patients. There was no correlation found between EBV and bacteria. However, the study found that 24% of the AD patients had two or more infections simultaneously, compared with 7.5% of the controls. This suggests that patients with AD are more susceptible to infections. However, the specific co-infection of bacteria and viruses was not established [63]. In their analysis of a different biofluid, S.A. Krynskiy et al. reported that, similar to saliva, higher EBV infectious rates were observed, along with elevated levels of EBV DNA, in individuals with MCI and AD compared to the control group [63].

### 3.4. Cytomegalovirus Infection and the Development of Dementia/Alzheimer’s Disease (AD)

The relationship between cytomegalovirus (CMV, or herpesvirus 5, HHV-5) infection and the development of dementia/Alzheimer’s disease (AD) is a subject of great interest and several articles, overall suggesting that CMV exposure may contribute to AD-related pathological processes.

#### 3.4.1. CMV and Amyloid-Related Biomarkers

Biomarker-based studies indicate that CMV seropositivity is primarily associated with alterations in amyloid metabolism. In particular, CMV seropositivity has been linked to lower plasma β-amyloid (Aβ) 1–42/β-amyloid 1–40 ratios, with higher anti-CMV IgG titers correlating with greater reductions in this ratio. No consistent data have been reported on alterations in levels of neurofilament light chain and glial fibrillary acidic protein [64,65]. This inverse relationship has been interpreted as a potential endogenous protective response, whereby Aβ production and aggregation may act as an antimicrobial defense against herpesvirus infection. CMV infection could promote central nervous system (CNS) Aβ production and accumulation, ultimately resulting in reduced peripheral Aβ42/Aβ40 ratios. A longitudinal analysis in cognitively unimpaired individuals has shown that herpesvirus seropositivity accelerates age-related changes in AD biomarkers. Lisa James and colleagues [37] demonstrated that the normalized rate of biomarker increase with age was more than twofold higher in herpesvirus-seropositive women compared with seronegative individuals. The strongest effects were observed for Aβ 1–40 and Aβ 1–42, whereas phosphorylated tau (pTau) species (pTau-181 and pTau-217) were less markedly affected. These associations were independent of APOE ε4 carrier status. Moreover, findings from a case–control study showed that CMV seropositivity has been reported to be significantly more prevalent in AD patients than in healthy controls, and co-positivity with other pathogens (such as *Chlamydia pneumoniae* and *Coxiella burnetii*) has been associated with faster clinical progression, poorer cognitive performance, and lower Aβ40/Aβ42 ratios [65].

#### 3.4.2. Conflicting Evidence on CMV Detection and Infection-Stage-Dependent Effects

It is important to note that some studies have not detected CMV in cohorts of Alzheimer’s patients studied [60,62]. However, proteomic analyses [53] suggest that the biological impact of CMV may differ depending on the immunological phase of infection. Higher CMV IgG levels in amyloid PET–positive individuals were associated with an earlier estimated age at amyloid accumulation onset, whereas CMV IgM levels was linked to: (I) a substantially reduced risk of dementia, (II) lower amyloid PET positivity, and (III) better performance on executive and verbal cognitive tasks. Despite these apparently protective associations, higher CMV IgG levels were still related to lower plasma Aβ42/Aβ40 ratios, reinforcing the notion of complex and stage-dependent interactions between CMV infection and amyloid pathology. Proteomic profiling further highlighted a prominent effect of CMV IgM on circulating proteins involved in neuroprotection and neuroplasticity, particularly immunoglobulin superfamily deleted in colorectal cancer subclass member 4 (IGDCC4).

The associations between CMV serology and peripheral amyloid biomarkers is not yet robust. Karin Lopatko Lindman and colleagues [24] found no significant correlations between anti-CMV IgG levels and plasma Aβ 1–42 or Aβ 1–40 concentrations in either AD patients or controls after correction for multiple comparisons. Some associations emerged in unadjusted analyses without a clear or reproducible pattern. The discrepancies may reflect the inherent limitations of plasma Aβ measurements, which do not necessarily mirror CNS amyloid burden and may fail to capture intracerebral pathogenic processes.

Beyond the amyloid process, accumulating evidence suggests that CMV infection may exert broader neurobiological effects through immune-mediated and neurotoxic mechanisms. CMV seroprevalence increased with age, supporting the hypothesis that chronic viral reactivation becomes more frequent in older individuals [21]. Indeed, age-related immune dysregulation, driven by sustained CMV-specific immune activation, may contribute to immunosenescence and low-grade chronic inflammation (“inflammaging”), a process that has also been linked to vascular dysfunction and other chronic conditions [21]. The CMV positivity association with age could be consistent with frequent reactivation or reinfection in later life [21].

#### 3.4.3. Systems Biology and Experimental Evidence of Neurotoxicity

Using a systems bioinformatics approach, Onisiforou et al. [52] demonstrated that CMV, along with HSV-1 and EBV, can modulate multiple core AD-related pathways, including oxidative stress, Aβ production, neuronal cell death, gliogenesis, and autophagy, via virus–host protein–protein interactions. Serum from CMV-seropositive cognitively healthy women has been shown to induce increased neuronal apoptosis in vitro and to be associated with poorer cognitive performance [68]. Moreover, CMV reactivation during SARS-CoV-2 infection was suggested to synergistically amplify AD risk by enhancing β-amyloid formation, oxidative stress, and autophagy dysregulation [52]. Convergent data from an experimental study in murine models further showed that the CMV immediate-early 2 (IE2) protein negatively affects cognition, promoting synaptic loss in the hippocampus and cerebral cortex, inducing disease-associated microglial activation, upregulating AD risk–related genes (including ApoE, Lpl, Cst7, Cd74, and Cd11c), and increasing amyloid plaque deposition and tau pathology [66]. Finally, analyses across multiple human cohorts have shown significantly elevated reactive oxygen species levels in AD patients with CMV or HSV-1 infection compared with AD patients without herpesvirus exposure, reinforcing the role of oxidative mechanisms in herpesvirus-associated neurodegeneration [67].

#### 3.4.4. CMV Seropositivity, Infection Burden, and Cognitive Performance

Regarding the impact on cognition, population-based studies have investigated the relationship between CMV seropositivity and cognitive performance, yielding mixed but informative results. In a large cohort of 5617 adults aged ≥65 years, CMV seropositivity was associated with poorer cognitive performance, although the association did not reach statistical significance overall. Notably, the magnitude of the association was greater among individuals with lower educational attainment, particularly those with less than a high school diploma, suggesting that limited cognitive reserve may exacerbate the cognitive impact of CMV infection [69]. Consistently, another study reported associations between CMV seropositivity and reduced executive function, as well as an increased incidence of mild cognitive impairment (MCI) and dementia [70]. Importantly, the relationship between CMV infection and cognitive outcomes appears to be modulated by age and genetic factors. In this context, carriers of the minor allele of the TNF-308 polymorphism (rs1800629) with higher CMV antibody levels exhibited better global cognitive performance, particularly in attention and processing speed domains. Furthermore, the positive association between CMV antibody levels and cognitive performance was more evident among individuals who did not carry the APOE ε4 allele, highlighting a complex interaction between immune response, genetic background, and cognitive trajectories [71].

Beyond single-pathogen exposure, the same evidence supports the hypothesis that cumulative infection burden with CMV involvement could be a relevant factor in cognitive impairment. A higher burden of microorganisms considered highly pathogenic for the brain was significantly associated with increased odds of MCI and dementia [22,72]. Partial contribution for a link between CMV and cognitive performance comes from studies by Wennberg et al. [39] and Stebbins et al. [73]. Both studies demonstrated that a greater number of positive pathogen-specific antibody tests, including CMV, was associated with worse performance on the Mini-Mental State Examination (MMSE). Specifically, the number of incorrectly answered MMSE items increased progressively as the number of positive antibody tests rose from zero to four, independent of demographic variables (such as sex, age, years of education, and comorbidity burden). Inflammatory markers appeared to play a contributory role, as higher circulating levels of interleukin-6 (IL-6) and tumor necrosis factor-α (TNF-α) were significantly associated with greater MMSE error rates. However, no significant relationship was observed between infection burden and incident dementia during follow-up, possibly due to the relatively small number of dementia cases in the cohorts studied [73].

#### 3.4.5. Longitudinal and Cohort Studies Reporting Null Associations

Vestin and colleagues [11] followed a large cohort of dementia-free individuals (mean age approximately 70 years) for 15 years and observed cumulative incidences of 4% for AD and 7% for all-cause dementia, where against all expectations, anti-CMV IgG seropositivity was not associated with an increased risk of either outcome. Similarly, Sithara Vivek et al. [74], studying elderly cancer survivors, found no significant association between CMV seropositivity and cognitive impairment. However, individuals who were both CMV seropositive and exhibited elevated inflammatory markers showed the highest odds of cognitive impairment, suggesting that inflammation may act as a critical mediator rather than CMV exposure alone. Comparable results were reported by Marques Zilli et al. [75], who analyzed CMV serology in 3048 individuals followed for 10 years and found no association between CMV seropositivity and dementia risk, total brain volume, or white matter hyperintensity burden on brain MRI. More recently, Ma et al. [76], analyzing UK Biobank data over a 15-year period, reported no association between CMV infection and incident AD, although this study was limited to White British participants with a relatively young mean age (56.7 years), raising concerns that age-related misalignment with typical AD onset may have attenuated detectable associations.

Overall, discrepancies among studies are likely attributable to differences in cohort age, duration of follow-up, and methodological design. In particular, studies involving older populations and shorter follow-up periods may be susceptible to reverse causality, whereby herpesvirus reactivation reflects early, preclinical neurodegenerative changes rather than acting as a primary causal factor. Additional limitations across studies include the lack of detailed information on infection severity, frequency of viral reactivation, and antiviral or immunomodulatory treatments, all of which may critically influence whether CMV exposure represents a risk or, under certain conditions, a modifying factor for cognitive decline. Based on the studies currently available, it is therefore not possible to identify a direct causal relationship between CMV infection and cognitive decline.

#### 3.4.6. Mechanistic Hypotheses: Molecular Mimicry and the Vagus Nerve

Among the hypotheses proposed to explain how CMV infection contributes to the development of cognitive impairment are molecular mimicry and the role of the vagus nerve. First, molecular mimicry between CMV-derived proteins and tau, in particular, a structural homology between CMV homologs and tau protein, may trigger immune cross-reactivity, thereby contributing to cerebral damage and to the development or progression of AD [77]. Analyses of chemical complementarity between T-cell receptor (TRA CDR3) sequences and CMV-derived peptides revealed that higher affinity for peptides from the CMV proteins UL131 and IRL4 was associated with higher Braak stages, suggesting a link between CMV-driven adaptive immune responses and tau pathology severity [77]. Second, more direct experimental evidence [78], that combined post-mortem immunohistochemical analyses with in vitro and organoid-based models to investigate the relationship between CMV infection, microglial activation and AD pathology, showed as patients that exhibiting CD38-positive microglia presented: increased IgG4 levels in the transverse colon, elevated IgG4 directed against CMV antigens in cerebrospinal fluid, and higher CMV and IgG4 signals in the superior frontal gyrus and vagus nerve. CMV exposure was sufficient (in vitro) to induce CD83 expression in microglia, a marker associated with antigen-presenting and immune-activated microglial states. Furthermore, infection of human cerebral organoids with CMV resulted in strong positive correlations between CMV abundance and both Aβ 1-42 and phosphorylated tau (pTau-212) levels. Based on these findings, the authors proposed that the vagus nerve may serve as a potential conduit for viral trafficking between the transverse colon and the brain, linking peripheral CMV infection, IgG4-mediated immune responses, and CD83-positive microglial activation in AD [78].

#### 3.4.7. CMV and Other Neurodegenerative Disorders

Evidence supporting a role for CMV in other neurodegenerative disorders is limited. In any case, a comparative analysis between patients with Parkinson’s disease and healthy controls revealed no significant differences in CMV antibody titers, nor any correlations between CMV serology and plasma α-synuclein levels, demographic characteristics, or clinical parameters [79]. No associations were observed between CMV antibody avidity and plasma α-synuclein concentrations or with polymorphisms in PILRA (paired immunoglobulin-like type 2 receptor alpha), an inhibitory receptor expressed on innate immune cells involved in the down-regulation of inflammatory responses. Although these findings do not fully exclude indirect or context-dependent effects, they argue against a direct involvement of CMV in the pathogenesis of Parkinson’s disease, highlighting a disease-specific interaction between CMV infection and neurodegenerative mechanisms that appears more relevant to AD than to synucleinopathies.

In conclusion, although this field arouses much interest from the perspective of prevention of cognitive impairment, it is plausible to believe, in light of the evidence reported, that further methodologically robust confirmations are needed to clearly define CMV infection as a risk factor for cognitive impairment and neurodegenerative disease.

### 3.5. Human Herpesvirus 6 and Alzheimer’s Disease

The literature linking human herpesvirus 6 (HHV-6) to Alzheimer dementia does not present a single uniform claim. Rather, it falls into several partially overlapping lines of inquiry that differ in what they treat as the key HHV-6-related phenomenon: direct molecular effects on canonical Alzheimer pathology, effects on amyloid biology, associations with cognition and dementia risk in human populations, preclinical biomarker shifts, evidence for viral presence in AD-relevant tissues, and broader contextual evidence from related neurodegenerative or neuroinflammatory settings. Taken together, these strands suggest that the HHV-6/AD relationship is best understood not as one hypothesis but as a set of mechanistic, epidemiologic, and interpretive questions.

#### 3.5.1. Direct Molecular Mechanisms Linking HHV-6 to Core AD Pathology

A first group of studies proposes that HHV-6 may contribute to Alzheimer disease through direct effects on molecular pathways central to neurodegeneration. Tang et al. present the most explicitly amyloid-centered mechanism, arguing that HHV-6A U4 interferes with ubiquitin-mediated proteasomal degradation of amyloid precursor protein (APP) by interacting with the DDB1/CUL4B E3 ubiquitin-ligase complex. In their model, this competition inhibits APP clearance, promotes APP accumulation, and favors downstream Aβ generation and deposition, thereby placing HHV-6A upstream of one of the defining lesions of AD [80]. By contrast, Hogestyn et al. focus less on amyloid itself and more on the consequences of viral latency. They show that the latency-associated HHV-6A transcript U94A impairs migration and cytoplasmic maturation in oligodendrocyte precursor cells and reduces neurite outgrowth and synapse maturation in human cortical neurons, supporting the idea that persistent latent HHV-6A products can destabilize cellular architecture and synaptic development in ways that may increase susceptibility to degeneration [81]. Romeo et al. add a third, inflammatory-stress dimension to this mechanistic literature by showing that HHV-6A infection in CNS cells dysregulates autophagy, induces ER stress and reactive oxygen species, activates STAT3, NF-κB, and mTOR signaling, and increases production of IL-6, IL-1β, CXCL13, and cathepsin S. In this formulation, HHV-6A is not only a potential amyloid-related trigger but also a driver of chronic cellular stress and neuroinflammatory signaling [82]. Thus, within the mechanistic literature, HHV-6 is variously positioned as a modulator of APP turnover, a latent disruptor of neuronal and glial structural integrity, and a promoter of inflammatory stress pathways relevant to AD pathogenesis.

#### 3.5.2. Amyloid Interaction and Plaque-Formation Hypothesis Tests

A second group of studies addresses a narrower but important question: whether roseolovirus infection can actually initiate or accelerate amyloid plaque formation. Bigley et al. provide the clearest test of this proposition. Using murine roseolovirus in the 5XFAD model, they show that the virus can infect the brain and induce transient neuroinflammation, and they further demonstrate that Aβ can bind viral particles and impair infection in vitro, a finding consistent with the antimicrobial-peptide view of amyloid. However, the central result of the study is negative: neither peripheral nor intracerebral infection accelerated Aβ plaque deposition in vivo. The same paper also extends the question to human tissue by analyzing brain RNA-seq data and concluding that human roseoloviruses were not overrepresented in AD brains. In subtype terms, this paper discusses human roseoloviruses broadly and specifically refers to HHV-6A and HHV-6B together, rather than isolating one subtype as the driver [83]. This line of work is therefore significant not because it dismisses all HHV-6-related effects, but because it narrows the field: it suggests that viral neuroinvasion, neuroinflammation, and even Aβ-virus interaction do not necessarily translate into enhanced plaque pathology. In literature-review terms, these studies function as an important counterweight to stronger causal formulations, showing that support for an HHV-6 role in AD does not automatically validate a simple amyloid-seeding model.

#### 3.5.3. Human Cognitive and Clinical Association Studies

A third line of work investigates whether HHV-6 is associated with measurable cognitive impairment or dementia-related clinical outcomes in human populations. Huang et al. provide one of the more directly relevant HHV-6-focused studies in this category. In an elderly community cohort, they report that HHV-6 not specified by subtype was not significantly associated with global cognitive impairment overall, but that higher HHV-6 copy number correlated with poorer performance in specific MMSE domains, namely orientation, attention and calculation, and language, with evidence of nonlinear dose–response relationships. This is important because it suggests that unspecified HHV-6 may relate more strongly to selective cognitive domains than to crude global case–control distinctions [84]. Broader epidemiological studies place this finding into a more qualified context. In UK Biobank, Mekli et al. found that dementia risk was most clearly associated with HSV-1 rather than HHV-6 alone, but that a combined seropositivity profile including HSV-1, HHV-6, HHV-7, and VZV was associated with increased dementia risk, implying that HHV-6 not specified by subtype may be better understood as part of a broader neurotropic viral burden than as an isolated exposure [18]. Similarly, Shi et al. report that HHV-6 not specified by subtype alone showed equivocal or no clear association with MCI-dementia, whereas cumulative infection burden across multiple pathogens was more consistently related to cognitive disorder risk [72]. Together, these studies suggest that the human clinical signal for HHV-6 is real but uneven: it appears strongest when framed in terms of domain-specific cognition or broader infectious burden, and weaker when HHV-6 is treated as a singular, independent determinant of dementia.

#### 3.5.4. Preclinical Biomarker and Risk-Stratification Studies

A fourth group of papers shifts the focus away from established dementia and toward earlier biological signatures of risk. James et al. examine cognitively unimpaired women and find that seropositivity for human herpesviruses is associated with steeper age-dependent increases in serum biomarkers linked to dementia, including Aβ40, Aβ42, pTau181, and pTau217. Here, the HHV-6 signal is explicitly serologic and combines HHV-6A and HHV-6B together, rather than distinguishing the two, placing HHV-6 within a preclinical framework in which viral exposure is associated not with diagnosed AD per se, but with biomarker trajectories consistent with heightened neurodegenerative vulnerability [37]. Duggan et al. extend this type of work in a more systems-level direction by relating antibody titers against common herpesviruses and coronaviruses to cognition, brain volumes, AD PET measures, plasma biomarkers, and a large plasma proteomic dataset. Their study identifies proteins such as IGDCC4 as potential mediators linking antiviral immune signatures to long-term neurodegenerative outcomes. In subtype terms, the paper analyzes herpesvirus antibody profiles at a broader panel level, so the HHV-6 component is best treated as HHV-6 not specified by subtype in the material available here [53]. Although HHV-6 is only one component of the broader viral panel, the conceptual move is important: these studies suggest that the HHV-6/AD question may not be exhausted by asking whether the virus is present in brain tissue. It may also depend on how host antiviral responses map onto early biomarker changes, systemic protein networks, and long-horizon neurodegenerative risk.

#### 3.5.5. Brain Detection and Pathogen-Presence Debates

Another important part of the literature concerns whether HHV-6 is actually enriched in AD-related tissues and how much evidentiary weight should be placed on detection studies. Bigley et al., again, are central here because their RNA-seq analysis argues against overrepresentation of HHV-6A or HHV-6B, challenging claims that detection in brain tissue is itself strong support for causation [83]. Nemergut et al. approach the issue from a broader multiplex-pathogen perspective, examining viral and bacterial pathogens in serum and CSF from AD patients and controls. Their strongest positive results concern *Treponema* spp. and the higher prevalence of multiple simultaneous infections in AD, rather than HHV-6 specifically. HHV-6 is included in the pathogen panel, but in the material available here, it is treated as HHV-6 not specified by subtype, and it is not the dominant discriminator between cases and controls [62]. The significance of this group is therefore largely interpretive: it shows that pathogen-detection findings may reflect background ubiquity, compartment-specific localization, or polymicrobial burden rather than a uniquely HHV-6-driven AD process. Accordingly, the presence-or-absence debate remains an important but limited part of the field, and by itself cannot resolve whether HHV-6 is causal, permissive, secondary, or incidental.

#### 3.5.6. Contextual and Indirect HHV-6 Neurodegeneration Evidence

Finally, a set of contextual studies does not directly test Alzheimer dementia but remains relevant because it establishes broader features of HHV-6 neurobiology that bear on AD plausibility. Jain et al. show that HHV-6 positivity in the *substantia nigra* of chronic alcoholics is associated with heightened microglial activation and more pronounced neuroinflammatory phenotypes, supporting the broader proposition that HHV-6 can amplify inflammatory injury within human brain tissue under pathological conditions. In the text available here, this paper discusses HHV-6 not specified by subtype [85]. Kasimir et al., though focused on ME/CFS rather than dementia, provide evidence for tissue-specific active HHV-6 signatures in postmortem neural tissues, reinforcing the idea that HHV-6 activity may be regionally restricted and not easily inferred from blood-based assays alone. Their framing explicitly includes HHV-6A and HHV-6B, although the paper is especially attentive to HHV-6 biology more broadly rather than isolating one subtype throughout [86]. Agostini et al., working in Parkinson’s disease, illustrate a related principle: herpesvirus-related neurodegenerative associations may depend on disease context, host immune response, and genetic background rather than on a uniform mechanism shared across all neurological disorders. However, that study is primarily about HSV-1, and for HHV-6, it only provides background comparator information rather than an AD-relevant HHV-6 mechanism; where HHV-6 appears, it is treated as HHV-6 not specified by subtype [79]. These studies are not AD evidence in a narrow sense, but they strengthen the conceptual backdrop against which AD-specific HHV-6 claims are interpreted, especially regarding persistence in neural tissue, conditional neuroinflammatory effects, and the possibility that host–virus interactions are disease-modifying rather than disease-specific.

Overall, the literature does not support a single conclusion so much as a layered picture. The strongest HHV-6-positive studies tend to support roles in APP regulation, cellular stress, latency-associated neural dysfunction, selective cognitive effects, and preclinical biomarker change, whereas the strongest cautionary studies challenge a simple model of HHV-6 overrepresentation in AD brain or straightforward acceleration of amyloid plaque formation. It seems, therefore, that HHV-6 remains a biologically plausible contributor to Alzheimer-related neurodegeneration, but one whose role is likely conditional, heterogeneous, and mediated through multiple partially independent pathways rather than a single universal mechanism.

### 3.6. HHV-7 and Alzheimer’s Disease

Building on the broader literature on infectious contributors to neurodegeneration, the specific evidence regarding HHV-7 and Alzheimer’s disease (AD) remains mixed and methodologically heterogeneous. Importantly, the three articles considered here address this question through distinct evidentiary frameworks and therefore should be read separately rather than collapsed into a single line of argument.

First, Nemergut et al. [62] address HHV-7 as a directly detectable pathogen in clinically relevant specimens. Using multiplex PCR, the authors examined serum and cerebrospinal fluid from AD patients and controls for a panel of viral and bacterial pathogens, including HHV-7. In this cohort, HHV-7 was not detected in any of the studied samples. This negative result is important because it constrains one possible interpretation of the infectious hypothesis: namely, that HHV-7 might be enriched in accessible peripheral or cerebrospinal compartments in AD. Although the study identified other microbial signals relevant to AD, including a higher frequency of *Treponema* spp. and multiple simultaneous infections, it does not provide evidence that HHV-7 is itself a detectable fluid-based correlate of AD. Thus, this article is best understood as offering negative evidence with respect to HHV-7 specifically, even while supporting a broader polymicrobial framework.

Second, Bigley et al. [83] address HHV-7 within the more mechanistic literature on roseoloviruses, amyloid biology, and AD neuropathogenesis. The authors explicitly situate their study in relation to prior reports suggesting associations between human roseoloviruses, especially HHV-6 and HHV-7, and AD. To test this hypothesis experimentally, they used murine roseolovirus as an in vivo surrogate for human roseoloviruses in the 5XFAD model. Their findings show that amyloid-β can interact with viral particles and that infection can induce neuroinflammation, but they do not show increased or accelerated amyloid plaque formation following infection. This is a critical transition in the argument, because it moves beyond observational association toward causal and mechanistic testing; on that question, the study does not support the proposition that roseolovirus infection drives amyloid deposition. Bigley et al. also extend their analysis to human brain tissue, thereby treating HHV-7 not only as a mechanistic candidate but also as a putative brain-resident virus that might be over-represented in AD. Using RNA-sequencing data from AD and control brains, they report no association between human roseolovirus infection and AD and specifically note that HHV-7 transcripts were not identified in either AD or non-AD control brains in their dataset. Accordingly, this article weighs against two related claims: first, that roseolovirus infection accelerates amyloid pathology in vivo, and second, that HHV-7 is detectably enriched in AD brain tissue. Its contribution to the literature is therefore primarily cautionary, insofar as it challenges stronger causal interpretations of earlier association studies.

Third, Mekli et al. [18] address HHV-7 from a population-level epidemiologic perspective by examining viral seropositivity and later dementia outcomes in the UK Biobank. This study differs conceptually from the other two because its relevant measure is not viral nucleic acid in tissue or fluid, but IgG seropositivity as an indicator of prior exposure. In analyses of individual viruses, only HSV-1 showed a statistically significant association with dementia risk. HHV-7 did not emerge as an independent signal. However, when the authors combined four neurotropic viruses—HSV-1, HHV-6, HHV-7, and VZV—seropositivity for all four was associated with increased dementia risk. The role of HHV-7 in this article is therefore indirect: it contributes to a broader cumulative exposure profile rather than standing out as a uniquely implicated agent. This distinction matters because the study supports a neurotropic viral burden model more readily than it supports a specific HHV-7 hypothesis.

Taken together, these articles suggest that the evidentiary status of HHV-7 in AD depends heavily on the level of analysis being used. Nemergut et al. test for direct pathogen detection in serum and CSF and find none; Bigley et al. test both mechanistic plausibility and brain-tissue representation and find no support for HHV-7 or roseoloviruses as drivers of amyloid pathology; and Mekli et al. examine prior viral exposure and show that HHV-7 may contribute only as one element within a broader neurotropic serologic profile. HHV-7 appears at present to be a candidate of limited and indirect support whose relevance, where present, appears to be contextual rather than independent.

### 3.7. HHV-8 and Alzheimer’s Disease

Recent systems-bioinformatics evidence supports a plausible mechanistic link between human herpesvirus 8 (HHV-8), also referred to as Kaposi sarcoma–associated herpesvirus (KSHV), and Alzheimer’s disease (AD). Onisiforou and Zanos [52] integrated experimentally validated virus–host protein–protein interactions with AD-associated molecular networks and found that KSHV ranked highest among infection-related KEGG pathways for centrality within the AD pathway-pathway network, suggesting a strong theoretical capacity to influence multiple AD-relevant pathways. When analyzed together with other Herpesviridae members, KSHV contributed to a shared set of 241 biological processes overlapping with AD, including pathways related to reactive oxygen species, autophagy dysregulation, amyloid-β formation, neuronal death, and gliogenesis. These findings are notable because each of these processes maps closely onto recognized features of AD pathogenesis, particularly oxidative stress, impaired proteostasis, and amyloid pathology. However, the study is computational and hypothesis-generating rather than confirmatory; it does not demonstrate that HHV-8 infection independently increases AD risk in humans, but instead positions HHV-8 as a biologically plausible contributor whose relevance warrants targeted experimental and epidemiologic investigation.

## 4. Discussion

The studies on the link between herpesviridae and AD published between December 2020 and December 2025 support an association but indicate that it is not uniform across viruses. Rather, the available evidence suggests that some herpesviruses are supported by a broader and more coherent body of data than others. Overall, HSV-1 appears to be the most consistently implicated virus, whereas VZV, CMV, EBV, and HHV-6 show variable but biologically plausible associations. By contrast, HHV-7 and HHV-8 currently appear to be supported by limited and mainly indirect evidence.

### 4.1. Integrated Critical Synthesis and Reasons for Discrepant Findings

The apparent inconsistency of the herpesvirus-AD literature is likely not random but reflects differences in how both viral exposure and Alzheimer-related outcomes are defined. Studies based on IgG seropositivity mainly capture lifetime exposure and cannot distinguish remote infection from repeated reactivation, whereas studies based on symptomatic infection, antiviral prescriptions, PCR, viral DNA, or post-mortem detection capture different biological phenomena. Therefore, null findings in serological cohorts do not necessarily contradict positive findings in studies of clinically overt or neurologically complicated infection; they may instead indicate that the timing, severity, anatomical compartment, and reactivation pattern of infection are more relevant than simple exposure status.

Population differences further contribute to divergent results. Associations may be stronger in older cohorts, APOE ε4 carriers, individuals with vascular comorbidity, or populations with reduced cognitive reserve, whereas younger cohorts or cohorts with short follow-up may be underpowered to detect AD outcomes. Outcome heterogeneity is equally important: all-cause dementia, clinically diagnosed AD, MCI, subjective cognitive decline, amyloid PET, plasma Aβ42/Aβ40, CSF tau, cortical thickness, and antidementia-drug dispensing are not interchangeable endpoints. This is particularly relevant for VZV and CMV, where vascular and inflammatory pathways may influence cognitive decline without necessarily producing a canonical AD biomarker profile.

Across independent cohorts, the most reproducible signal concerns HSV-1, especially when exposure is defined by clinically meaningful infection or when biomarker and neuroimaging endpoints are considered together. VZV shows a more indirect but clinically relevant pattern, driven by studies of zoster, neurological complications, and vaccination. By contrast, EBV, CMV, HHV-6, HHV-7, and HHV-8 currently provide more fragmented evidence, with several mechanistic or biomarker observations but less consistent longitudinal clinical confirmation.

For interpretive clarity, the evidence was considered along a causal-inference gradient rather than as a single homogeneous body of data. Longitudinal human cohorts, quasi-experimental vaccination analyses, and randomized or interventional studies were considered most informative for clinical inference when temporality and confounding control were adequate. Mendelian randomization studies were interpreted as potentially informative but dependent on the validity of genetic instruments, absence of pleiotropy, and careful separation of immune response from infection itself. Cross-sectional serological studies, post-mortem analyses, and biomarker studies were treated as association-level evidence because they cannot reliably establish temporal direction. Animal, organoid, in vitro, and in silico studies were interpreted as mechanistic or hypothesis-generating evidence rather than direct proof of clinical causality. This framework was used throughout the discussion to reduce biological overinterpretation and to distinguish association from causation.

### 4.2. Hierarchy of Evidence and Causal Interpretability

This hierarchy was used to avoid treating all evidence streams as equivalent. Longitudinal human studies with clearly antecedent exposure and adjudicated cognitive outcomes provide stronger support for association than cross-sectional serological comparisons. Natural-experiment and vaccination studies are particularly informative because they test whether modification of herpesvirus-related risk is followed by altered dementia incidence, although they remain vulnerable to residual confounding by healthcare behavior, socioeconomic status, baseline frailty, and vaccine eligibility criteria. Mendelian randomization may strengthen causal interpretation, but only when instruments are specific and pleiotropy is unlikely.

Mechanistic studies in animals, organoids, post-mortem tissue, in vitro systems, and in silico models occupy a different position in the evidentiary hierarchy. They are valuable because they identify candidate biological pathways, such as amyloid dysregulation, tau phosphorylation, neuroinflammation, vascular injury, immune activation, molecular mimicry, and oxidative stress. However, these findings mainly establish plausibility and cannot by themselves demonstrate that a given herpesvirus initiates or accelerates AD in humans. Causal claims therefore require convergence between mechanistic plausibility and reproducible longitudinal or interventional human evidence.

Among all herpesviridae, HSV-1 appears to be the most relevant in relation to AD. This appears to be supported by the fact that HSV-1 is the virus for which the literature offers the greatest convergence of epidemiological, biomarker, neuroimaging, and mechanistic findings. Several cohort and case–control studies report an association between HSV-1 infection or seropositivity and increased risk of dementia or AD, in some cases with stronger associations among older individuals and APOE ε4 carriers. In parallel, imaging and biomarker studies indicate that HSV-1 exposure may be associated with hippocampal atrophy and greater cerebral amyloid burden, even in cognitively normal individuals. Moreover, experimental studies suggest that HSV-1 may influence amyloid-β accumulation, tau phosphorylation, and neuroinflammatory pathways. However, these findings are not entirely consistent, since several neuropathological and animal studies failed to confirm a direct role of HSV-1 in accelerating amyloid plaque formation or in colocalizing with AD neuropathological lesions. Hence, the relevance of HSV-1 appears substantial, but not definitively established.

The recent evidence on the association between HSV-1 and AD reviewed here is indeed still affected by the issue of contrasting effects present also in previous studies on the topic. Indeed, some previous reports suggest that HSV-1 seropositivity may reduce the risk of progression from mild cognitive impairment to AD [93,94], and population-based studies failed to confirm a direct association with AD [95].

VZV also emerges as a potentially relevant virus, although its putative contribution to AD seems to differ from that proposed for HSV-1. In particular, the VZV literature appears to emphasize vascular, inflammatory, and systemic mechanisms more than direct neuronal amyloid-related effects. Mechanistic studies indicate that VZV infection may promote amyloidogenic and tau-related changes in vascular-associated brain cells, whereas epidemiological studies suggest that herpes zoster, especially when severe or involving the central nervous system, may be associated with increased subsequent risk of dementia. This appears particularly relevant in light of the vaccination studies, which repeatedly show that zoster vaccination is associated with lower dementia risk. However, such findings should be interpreted with caution, since vaccine-associated protection may also reflect differences in healthcare access, health-seeking behavior, or broader immune effects rather than a purely VZV-specific mechanism. Therefore, VZV appears to be an important candidate, although its role may be mediated more by neuroinflammation and cerebrovascular injury than by direct induction of canonical AD pathology.

The evidence regarding CMV is more heterogeneous. On the one hand, CMV seropositivity and higher antibody titers have been associated with altered amyloid-related biomarkers, particularly lower plasma Aβ42/Aβ40 ratios, and some experimental studies suggest that CMV-related proteins may promote synaptic injury, microglial activation, amyloid deposition, and tau-related changes. On the other hand, several large longitudinal studies did not find clear associations between CMV seropositivity and incident AD or dementia. This discrepancy suggests that CMV may not act as an independent determinant of AD, but rather as a factor that modifies neurodegenerative risk under particular conditions, such as immunosenescence, chronic inflammation, or advanced age. In this sense, CMV appears biologically plausible, but its epidemiological relevance remains uncertain.

A similarly mixed picture emerges for EBV. The relevance of EBV is supported by several Mendelian randomization studies, which suggest that genetically predicted immune responses related to EBV may be associated with increased AD risk. In addition, serological and proteomic studies indicate associations between EBV-related immune markers and amyloid burden, brain structure, and dementia-related biomarkers. Experimental and in silico studies also propose plausible mechanisms involving ApoE interaction, amyloid-like aggregation, and pro-inflammatory activation. Nevertheless, the EBV literature remains less consistent than that of HSV-1, and much of the available evidence concerns host immune responses rather than direct demonstration of EBV activity in the brain. Thus, EBV appears relevant mainly within a broader framework of chronic immune activation and host–virus interaction, rather than as a clearly established direct contributor to AD neuropathology.

The findings concerning HHV-6 also support biological plausibility, but not a clearly defined independent association with AD. In particular, mechanistic studies suggest that HHV-6A may interfere with APP degradation, induce oxidative and endoplasmic reticulum stress, dysregulate autophagy, and impair neural and glial maturation. These observations are of interest because they link HHV-6 to several core pathways involved in neurodegeneration. However, human studies provide weaker support. Clinical and epidemiological investigations more often show associations with selected cognitive domains, preclinical biomarker trajectories, or cumulative infectious burden than with AD diagnosis itself. In addition, the frequent lack of distinction between HHV-6A and HHV-6B limits interpretation. Therefore, HHV-6 appears to be a plausible but still incompletely characterized contributor.

By contrast, the current evidence suggests that HHV-7 has limited relevance as an independent factor in AD. The studies reviewed do not support a clear direct association between HHV-7 and AD-related pathology, cognition, or brain tissue involvement. Negative findings in CSF and serum studies, as well as in experimental roseolovirus models and transcriptomic analyses of brain tissue, argue against a major direct role. HHV-7 appears associated with dementia risk mainly within combined neurotropic viral exposure profiles rather than as a stand-alone factor. Hence, HHV-7 appears at present to have, at most, a contextual role within cumulative infectious burden.

An even more cautious interpretation is required for HHV-8, for which the available evidence is almost entirely computational. Systems-bioinformatics analyses indicate that HHV-8 may in principle intersect with AD-related pathways such as oxidative stress, autophagy dysregulation, amyloid formation, neuronal death, and gliogenesis. However, no direct epidemiological, neuropathological, or experimental evidence was identified showing that HHV-8 infection is associated with AD risk or pathology in humans. Therefore, HHV-8 should currently be regarded as a hypothesis-generating candidate rather than a supported etiological factor.

### 4.3. Caution in Interpreting Mechanistic and in Silico Findings

Mechanistic and computational studies are indispensable for generating testable hypotheses, but their translational meaning should remain circumscribed. Molecular docking, pathway enrichment, protein-interaction prediction, and in silico overlap analyses can identify candidate mechanisms, yet they do not establish infection, temporal precedence, dose–response relationships, or disease specificity in humans. Similarly, organoid and animal models can reproduce selected AD-like molecular phenotypes but cannot fully model decades-long latency, intermittent reactivation, aging-related immune remodeling, vascular comorbidity, or mixed dementia pathology.

Accordingly, the EBV and HHV-8 sections should be interpreted as evidence of biological plausibility rather than direct clinical causation. The same caution applies to HHV-6 and CMV mechanistic findings unless they are supported by consistent human longitudinal, biomarker, or interventional data.

**Figure 2:**
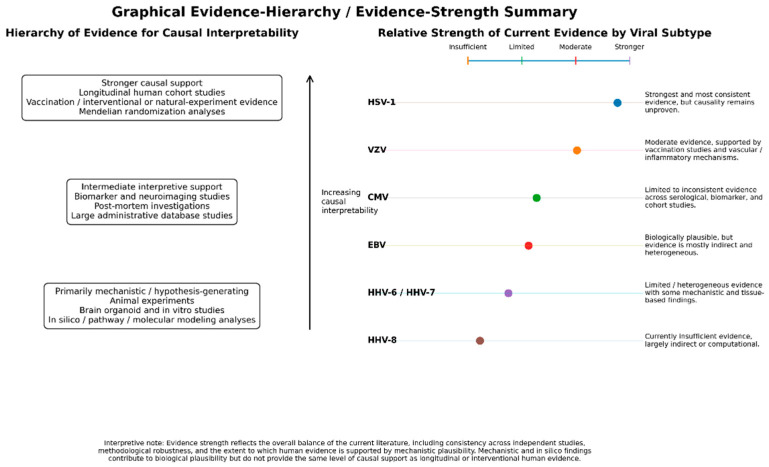
Qualitative evidence hierarchy and relative strength of evidence across herpesvirus subtypes.

Taken together, the literature suggests an approximate gradient of evidentiary support among herpesviridae in AD (Figure 2). HSV-1 is the most extensively studied and most consistently supported candidate, with convergent but still non-definitive evidence from epidemiological, biomarker, neuroimaging, and mechanistic studies. VZV has substantial but partly indirect support, particularly from vascular, inflammatory, and vaccination-related studies. CMV, EBV, and HHV-6 occupy an intermediate position characterized by biological plausibility and selected positive findings but inconsistent human evidence. HHV-7 and HHV-8 remain weakly supported, with evidence that is limited, indirect, or primarily mechanistic. This hierarchy should not be interpreted as proof of causality; it reflects both biological plausibility and the uneven quantity and quality of available studies.

Indeed, one major issue is the possibility of study bias related to uneven scientific attention. HSV-1 has long occupied a central place in the infectious hypothesis of AD, and this likely contributed to a greater number of studies, a wider range of methodological approaches, and a more developed mechanistic framework. Consequently, HSV-1 may appear more relevant in part because it has been examined more thoroughly than other herpesviruses. Conversely, viruses such as HHV-7 and HHV-8 may appear less important, not necessarily because they are biologically irrelevant, but because they have been investigated less often and with less methodological depth. A similar problem applies to HHV-6, for which insufficient subtype-specific analyses may have obscured meaningful associations.

Another possible source of distortion is related to viral frequency and detectability. Highly prevalent viruses such as HSV-1, CMV, EBV, and VZV are easier to study in large cohorts because seropositivity is common, but their ubiquity also makes causal inference more difficult. At the same time, severe or clinically overt infections, such as herpes zoster with neurological complications or symptomatic HSV infection recorded in health databases, may show stronger associations because they capture more intense or clinically recognizable viral activity. Therefore, the literature may be influenced both by the ease with which common viruses can be measured and by the greater visibility of severe infections, whereas latent, tissue-restricted, or less frequently diagnosed infections may remain underrepresented.

Interpretation is further complicated by the heterogeneity of the available evidence. The reviewed studies include serological investigations, retrospective health-record analyses, Mendelian randomization studies, neuropathological analyses, in vitro and organoid experiments, animal models, and computational approaches. These forms of evidence are not equivalent. Serological studies are often unable to distinguish remote exposure from reactivation or persistent immune stimulation. Retrospective observational studies are vulnerable to confounding and reverse causation. Experimental models are useful for mechanistic testing, but may not adequately reproduce latency, aging, repeated reactivation, or the complexity of human brain pathology. For this reason, the evidence should not be interpreted as uniformly strong across viruses or across study designs.

A further limitation concerns outcome heterogeneity. Some studies focus on clinically diagnosed AD, others on all-cause dementia, subjective cognitive decline, mild cognitive impairment, MMSE performance, amyloid PET, plasma biomarkers, or use of antidementia medication. These outcomes are not interchangeable and may reflect partially distinct processes. This appears particularly relevant for VZV and CMV, whose putative effects may be mediated more through vascular or inflammatory pathways than through direct promotion of classical AD lesions. Therefore, combining these outcomes under a single AD framework may overestimate specificity.

Finally, the possibility of reverse causation cannot be excluded. Older individuals with preclinical neurodegeneration may be more susceptible to infection, more likely to experience viral reactivation, or more likely to have clinically recognized and recorded infections. In such cases, herpesvirus infection or reactivation might represent a marker of frailty, immune dysfunction, or ongoing neurodegeneration rather than a primary causal factor.

### 4.4. Reverse Causation and Directionality

Directionality is one of the most important unresolved questions in this literature. Preclinical AD is accompanied by changes in innate and adaptive immunity, neuroinflammation, blood–brain barrier function, glymphatic clearance, and systemic frailty. These changes could reduce antiviral surveillance and increase susceptibility to herpesvirus reactivation. Thus, a positive association between herpesvirus markers and AD may reflect infection contributing to neurodegeneration, but it may also reflect viral reactivation secondary to emerging neurodegenerative disease.

Reverse causation is especially relevant for cross-sectional studies, post-mortem viral detection, and retrospective health-record analyses in which the temporal relationship between infection, reactivation, biomarker abnormality, and cognitive decline cannot be firmly established. Neurodegenerative tissue damage could also increase the detectability or persistence of viral nucleic acids without proving that the virus initiated the pathological process. For this reason, positive retrospective and cross-sectional findings should be interpreted as signals of association and biological relevance rather than as proof of etiological causation.

### 4.5. Overall Assessment by Viral Subtype

HSV-1 currently has the strongest and most consistent evidence base among the herpesviridae reviewed, although causality remains unproven. VZV has moderate evidence, particularly because of vaccination studies and vascular-inflammatory mechanisms. CMV shows biologically plausible but inconsistent evidence, with several null longitudinal studies. EBV remains plausible but largely indirect, with important contributions from immune-response, MR, and molecular studies but limited direct clinical evidence. HHV-6 and HHV-7 have limited and heterogeneous support, whereas HHV-8 is currently supported only by indirect computational evidence and should be considered insufficiently established in relation to AD.

In conclusion, the available literature suggests that herpesviridae may be associated with AD through multiple partially overlapping pathways, including amyloid and tau dysregulation, chronic neuroinflammation, vascular injury, immune dysregulation, and cumulative infectious burden. Among the viruses reviewed, HSV-1 appears to be the most relevant candidate, with VZV also supported by a substantial, though partly indirect, body of evidence. For CMV, EBV, and HHV-6, the evidence remains biologically plausible but inconsistent, while HHV-7 and HHV-8 are supported only by limited or indirect data. Importantly, the current literature does not establish a direct causal relationship between any individual herpesvirus and AD. Methodological heterogeneity, residual confounding, exposure misclassification, unequal study intensity across viruses, and possible reverse causation remain major limitations. Future research should prioritize longitudinal cohorts with subtype-specific viral assessment, repeated measures of reactivation, standardized AD biomarker and clinical outcomes, careful confounder control, and intervention-oriented designs capable of distinguishing causal contribution from bystander immune activation or disease-related susceptibility to infection.

## Figures and Tables

**Table 1:** Structured appraisal framework used to interpret the relative strengths and limitations of the included evidence.

Main Interpretive Limitations	Sample Size and Confounder Control	Structured Appraisal Features	Evidence Domain
Residual confounding, exposure misclassification, long latency, and incomplete reactivation data	Usually medium to large with follow-up; age/sex/comorbidity adjustment common, APOE variably included	Mostly serology or recorded clinical infection; outcomes include incident AD, dementia, cognitive decline, or biomarkers	Prospective population cohorts
Healthcare-seeking bias, coding error, limited biological specificity, and unclear temporality	Often very large; adjustment may be extensive but limited to coded variables	ICD or prescription-based proxies for clinically recognized infection; outcomes include coded dementia/AD or medication dispensing	Administrative database studies
Validity depends on instrument specificity, pleiotropy assumptions, and phenotype definition	Large genetic datasets; conventional confounding partly reduced	Genetically proxied immune response or infection-related traits linked to AD or dementia endpoints	Mendelian randomization
Cross-sectional design, multiple testing, and uncertainty about whether biomarker shifts predict clinical AD	Usually small to moderate; APOE and age adjustment not uniform	Serology, PCR, or viral DNA/RNA detection linked to amyloid/tau biomarkers, PET, CSF/plasma markers, cortical thickness, or hippocampal volume	Biomarker and neuroimaging studies
High mechanistic value but limited direct causal inference for human AD	Small experimental or computational datasets; experimental control possible, but clinical confounding not addressed	Direct infection, tissue viral detection, pathway modeling, or protein-interaction prediction linked to molecular pathways, inflammation, amyloid/tau change, or neurotoxicity	Post-mortem, animal, organoid, in vitro, and in silico studies

**Table 2:** Classification of included studies according to virus, inferential level, outcome domain, and model system.

Virus	Study (Author, Year, Reference)	Association vs. Causation	Biomarkers vs. Clinical Outcome	Human vs. Animal Model
HSV-1/2	Vestin et al., 2024 [11]	Association	Clinical Outcome (Dementia Risk)	Human
HSV-1/2	Linard et al., 2021 [12]	Association	Biomarker (Hippocampal Volume) + Clinical Outcome (AD)	Human
HSV-1	Liu et al., 2025 [13]	Association	Clinical Outcome (AD Risk)	Human
HSV-1/2	Shim et al., 2022 [14]	Association	Clinical Outcome (Dementia Incidence)	Human
HSV-1/2	Araya et al., 2025 [15]	Association	Clinical Outcome (Dementia Risk)	Human
HSV-1/2	Shin et al., 2024 [16]	Association	Clinical Outcome (Dementia Risk)	Human
HSV-1	Cantero et al., 2024 [17]	Association	Biomarker (Cerebral Aβ Load)	Human
HSV-1/VZV/EBV	Mekli et al., 2022 [18]	Association	Clinical Outcome (Dementia)	Human
HSV-1	Tejeda et al., 2023 [19]	Association	Clinical Outcome (AD Risk) + Biomarker (Viral DNA)	Human
HSV-1/2	Beydoun et al., 2024 [20]	Association	Clinical Outcome (AD Dementia)	Human
HSV-1	Olsson et al., 2024 [21]	Association (Hypothesis)	Clinical Outcome (AD Pathogenesis Implications)	Human
HSV-1/2	Rangel et al., 2025 [22]	Association	Clinical Outcome (Cognitive Impairment)	Human
HSV-2	Roberts et al., 2023 [23]	Association	Biomarker (Cortical Thickness)	Human
HSV-1/CMV	Lopatko Lindman et al., 2021 [24]	Association (Null)	Biomarker (Aβ40/Aβ42 Levels)	Human
HSV-1	Lapeyre et al., 2024 [25]	Causation (Experimental)	Biomarker (Aβ Plaque Load)	Animal (APP/PS1 Mice)
HSV-1	Bocharova et al., 2022 [26]	Causation (Experimental)	Biomarker (Aβ Aggregation, Immune Response)	Animal (5XFAD, hAβ/APOE4 Mice)
HSV-1	Tran et al., 2021 [27]	Causation (Observational Post-Mortem)	Biomarker (Aβ, pTau Expression)	Human (Post-Mortem)
HSV-1	Bocharova et al., 2021 [28]	Causation (Experimental)	Biomarker (Aβ Localization, Viral Replication)	Animal (5XFAD Mice)
HSV-1/2	Devanand et al., 2026 [29]	Causation (Intervention)	Clinical Outcome (Cognitive Decline) + Biomarkers (AD)	Human
HSV-1/2/VZV	Tan et al., 2024 [30]	Association	Clinical Outcome (Dementia Medication Dispensing)	Human
HSV-1	Sundstrom et al., 2024 [31]	Causation (Experimental)	Biomarker (Inflammation, Gene Expression)	Human (Brain Organoids)
HSV-1	Qiao et al., 2022 [32]	Causation (Experimental)	Biomarker (Aβ-Associated Neuropathology)	Human (Brain Organoids)
VZV	Bubak et al., 2020 [33]	Causation (Mechanistic)	Biomarker (CSF amyloid, amylin, anti-VZV antibodies)	Human (In vitro)
VZV	Bubak et al., 2022 [34]	Causation (Mechanistic)	Biomarker (Amyloidogenic peptides, intracellular amyloid)	Human (In vitro)
VZV	Shinomoto et al., 2021 [35]	Association	Biomarker (CSF profiles mimicking AD)	Human
VZV	Eckerström et al., 2020 [36]	Association (Null)	Biomarker (CSF AD pathology)	Human
HSV-1/VZV/EBV/CMV	James et al., 2025 [37]	Association	Biomarker (Serum Aβ40, Aβ42, pTau181, pTau217)	Human
VZV	Lophatananon et al., 2021 [38]	Association (Null)	Clinical Outcome (Dementia risk)	Human
VZV	Wennberg et al., 2023 [39]	Association	Clinical Outcome (Cognitive performance)	Human
VZV	Choi et al., 2021 [40]	Association (Null)	Clinical Outcome (Neurodegenerative dementia)	Human
VZV	Yeh et al., 2024 [41]	Association	Clinical Outcome (Subjective cognitive decline)	Human
VZV	Lehrer & Rheinstein, 2021 [42]	Association	Clinical Outcome (Dementia risk)	Human
VZV	Lophatananon et al., 2023 [43]	Causation (Intervention)	Clinical Outcome (Dementia risk)	Human
VZV	Xie et al., 2025 [44]	Causation (Intervention)	Clinical Outcome (Dementia, MCI, deaths due to dementia)	Human
VZV	Harris et al., 2023 [45]	Association	Clinical Outcome (AD risk)	Human
VZV	Taheri et al., 2025 [46]	Association	Biomarker (Cytokine expression: TNFα, IFNγ, TGFβ, IL-10)	Human
EBV	Zhang et al., 2025 [47]	Causation (Mendelian Randomization)	Clinical Outcome (AD risk)	Human
EBV	Huang et al., 2021 [48]	Causation (Mendelian Randomization)	Clinical Outcome (AD risk)	Human
EBV	Peng et al., 2025 [49]	Causation (Mendelian Randomization)	Clinical Outcome (AD risk)	Human
EBV	Carbone et al., 2014 [50]	Association	Clinical Outcome (Cognitive decline)	Human
EBV	Sim et al., 2024 [51]	Association	Clinical Outcome (AD risk)	Human
EBV/HHV-8	Onisiforou & Zanos, 2024 [52]	Causation (Mechanistic, In silico)	Biomarker (AD pathways: ROS, autophagy, Aβ formation)	In silico
EBV	Duggan et al., 2025 [53]	Association	Biomarker (Aβ42/40 ratio, cortical amyloid, frontal lobe volume)	Human
EBV	Murdock et al., 2022 [54]	Association (Null)	Biomarker (Executive function, inflammation)	Human
EBV	Tiwari et al., 2022 [55]	Causation (Mechanistic, In silico)	Biomarker (ApoE interaction, amyloid-b binding)	In silico
EBV	Tiwari et al., 2021 [56]	Causation (Mechanistic, In silico)	Biomarker (Amyloid-like aggregates, cytotoxicity)	In silico
EBV	Patra et al., 2023 [57]	Causation (Mechanistic, In vitro)	Biomarker (Pro-inflammatory cytokines: IL-1β, IL-6, TNF-α)	In vitro
EBV	Tiwari et al., 2022 [58]	Causation (Mechanistic, In silico)	Biomarker (EBV-dUTPase inhibition)	In silico
EBV	Awasthi et al., 2024 [59]	Causation (Mechanistic, In silico)	Biomarker (miRNAs, hub genes: YWHAH, YWHAG, etc.)	In silico
EBV	Malashenkova et al., 2021 [60]	Association	Clinical Outcome (AD clinical parameters)	Human
EBV	Levine et al., 2023 [61]	Association	Clinical Outcome (Dementia)	Human
EBV/HHV-6/HHV-7	Nemergut et al., 2022 [62]	Association (Null)	Biomarker (EBV/HHV-6/HHV-7 DNA in serum/CSF/brain)	Human
EBV	Krynskiy et al., 2021 [63]	Association	Biomarker (EBV DNA in MCI/AD)	Human
CMV	Parker et al., 2024 [64]	Association	Biomarker (Plasma Aβ42/Aβ40 ratio)	Human
CMV	Garcia-Bustos et al., 2025 [65]	Association	Biomarker (Plasma Aβ42/Aβ40 ratio) + Clinical Outcome (Cognitive performance)	Human
CMV	Liu et al., 2023 [66]	Causation (Experimental)	Biomarker (Amyloid plaque deposition, tau pathology)	Animal (Murine)
CMV	Khodamoradi et al., 2025 [67]	Association	Biomarker (Reactive oxygen species)	Human
CMV	James et al., 2024 [68]	Causation (Experimental)	Biomarker (Neuronal apoptosis)	In vitro
CMV	Stebbins et al., 2020 [69]	Association	Clinical Outcome (Cognitive performance)	Human
CMV	Roberts et al., 2025 [70]	Association	Clinical Outcome (MCI, dementia)	Human
CMV	Vacher et al., 2025 [71]	Association	Clinical Outcome (Cognitive performance)	Human
CMV/HHV-6	Shi et al., 2024 [72]	Association	Clinical Outcome (MCI, dementia)	Human
CMV	Stebbins et al., 2021 [73]	Association	Clinical Outcome (MMSE performance)	Human
HSV-1/2/CMV	Vestin et al., 2024 [11]	Association (Null for CMV, Association for HSV-1/2)	Clinical Outcome (Dementia Risk/AD, all-cause dementia)	Human
CMV	Vivek et al., 2021 [74]	Association (Null)	Clinical Outcome (Cognitive impairment)	Human
CMV	Zilli et al., 2021 [75]	Association (Null)	Clinical Outcome (Dementia)	Human
CMV	Ma et al., 2024 [76]	Association (Null)	Clinical Outcome (AD)	Human
CMV	Blanck et al., 2023 [77]	Causation (Mechanistic, Hypothesis)	Biomarker (Tau, molecular mimicry)	Human (Hypothesis)
CMV	Readhead et al., 2024 [78]	Causation (Experimental)	Biomarker (Aβ1-42, pTau-212, CD83+ microglia)	Human (Post-mortem) + In vitro + Organoids
CMV	Agostini et al., 2021 [79]	Association (Null)	Biomarker (α-synuclein)	Human
HHV-6	Tang et al., 2022 [80]	Causation (Mechanistic, In vitro)	Biomarker (APP accumulation, Aβ generation)	In vitro
HHV-6	Hogestyn et al., 2022 [81]	Causation (Mechanistic, In vitro)	Biomarker (Cytoskeletal dysfunction)	In vitro
HHV-6	Romeo et al., 2022 [82]	Causation (Mechanistic, In vitro)	Biomarker (Inflammatory cytokines, ER stress)	In vitro
HHV-6/HHV-7	Bigley et al., 2022 [83]	Causation (Experimental)	Biomarker (Aβ pathology)	Animal (Murine) + Human (Brain tissue)
HHV-6	Huang et al., 2022 [84]	Association	Clinical Outcome (Cognitive performance)	Human
EBV/HHV-6	Duggan et al., 2025 [53]	Association	Biomarker (Plasma proteome)	Human
HHV-6	Jain et al., 2021 [85]	Association	Biomarker (Microglial activation)	Human (Post-mortem)
HHV-6	Kasimir et al., 2022 [86]	Association	Biomarker (Active HHV-6 signatures)	Human (Post-mortem)

## Data Availability

No new data were created or analyzed in this study. Data sharing does not apply to this article.

## Abbreviations

The following abbreviations are used in this manuscript:

Aβ	Amyloid beta
AD	Alzheimer’s disease
ApoE/APOE	Apolipoprotein E/apolipoprotein E gene
APP	Amyloid precursor protein
BRFSS	Behavioral Risk Factor Surveillance System
BZLF-1	BamHI Z Epstein–Barr virus leftward frame 1
CMV	Cytomegalovirus
CNS	Central nervous system
CPRD	Clinical Practice Research Datalink
CSF	Cerebrospinal fluid
DHE	Dehydroevodiamine
DNA	Deoxyribonucleic acid
EBNA-1	Epstein–Barr virus nuclear antigen 1
EBV	Epstein–Barr virus
ELISA	Enzyme-linked immunosorbent assay
ER	Endoplasmic reticulum
HCMV	Human cytomegalovirus
HHV	Human herpesvirus
HHV-3	Human herpesvirus 3/varicella-zoster virus
HHV-5	Human herpesvirus 5/cytomegalovirus
HHV-6	Human herpesvirus 6
HHV-6A	Human herpesvirus 6A
HHV-6B	Human herpesvirus 6B
HHV-7	Human herpesvirus 7
HHV-8	Human herpesvirus 8
HSE	Herpes simplex encephalitis
HSV	Herpes simplex virus
HSV-1	Herpes simplex virus type 1
HSV-2	Herpes simplex virus type 2
ICD	International Classification of Diseases
IDE	Insulin-degrading enzyme
IgG	Immunoglobulin G
IgM	Immunoglobulin M
IL	Interleukin
IL-1β	Interleukin-1 beta
IL-6	Interleukin-6
IL-10	Interleukin-10
IRL4	Internal repeat long 4
KEGG	Kyoto Encyclopedia of Genes and Genomes
KSHV	Kaposi sarcoma-associated herpesvirus
LPS	Lipopolysaccharide
MCI	Mild cognitive impairment
ME/CFS	Myalgic encephalomyelitis/chronic fatigue syndrome
MMSE	Mini-Mental State Examination
MR	Mendelian randomization
MRI	Magnetic resonance imaging
MS	Multiple sclerosis
NF-κB	Nuclear factor kappa-light-chain-enhancer of activated B cells
NHANES	National Health and Nutrition Examination Survey
NOMAS	Northern Manhattan Study
PCR	Polymerase chain reaction
PET	Positron emission tomography
PICOS	Population, Intervention/Exposure, Comparator, Outcomes, Study design
PILRA	Paired immunoglobulin-like type 2 receptor alpha
PRISMA	Preferred Reporting Items for Systematic Reviews and Meta-Analyses
pTau	Phosphorylated tau
RNA	Ribonucleic acid
RNA-seq	RNA sequencing
ROS	Reactive oxygen species
SARS-CoV-2	Severe acute respiratory syndrome coronavirus 2
SCD	Subjective cognitive decline
STAT3	Signal transducer and activator of transcription 3
Tdap/Td	Tetanus, diphtheria, and pertussis/tetanus and diphtheria vaccine
TGF-β	Transforming growth factor beta
TNF-α	Tumor necrosis factor alpha
VCA	Viral capsid antigen
VZV	Varicella-zoster virus
WES	Whole-exome sequencing
WGS	Whole-genome sequencing
WOS	Web of Science
